# Factors related with quality on sleep of daytime workers

**DOI:** 10.1186/s40557-018-0271-7

**Published:** 2018-10-23

**Authors:** Bu-il Kim, Seong-yong Yoon, Jin-seok Kim, Kuck-Hyeun Woo, Seong-yong Cho, Ho Lee, Jong-min An

**Affiliations:** Department of Occupational and Environmental Medicine, Soonchunhyang University Gumi Hospital, 179 1gongdan-ro, Gumi, Gyeongsangbuk-do Republic of Korea

**Keywords:** Sleep, Daytime workers, Sleep quality, Sleep duration

## Abstract

**Background:**

This study was conducted to identify the sleep status of daytime workers who do not work in shifts. This study analyzed factors affecting sleep duration and sleep quality.

**Methods:**

This study was conducted on 1171 daytime workers at a manufacturing workshop. We used a self-administered questionnaire to investigate demographic variables, work type, working period, musculoskeletal symptoms and the Pittsburgh Sleep Quality Index to assess sleep. Regular health checkup was conducted for the worker’s clinical examination.

**Results:**

The mean sleep duration was 6.36 h and the mean score on the Pittsburgh Sleep Quality Index was 4.46. Work type and obesity were related to sleep duration. Age, obesity and musculoskeletal pain were significantly related to sleep quality. The prevalence ratio of researcher group for short sleep duration was 1.27 (95% confidence interval: 1.02–1.58). The prevalence ratio of those aged 50 years and over was 0.47 (0.25–0.91) and of those in their 40s was 0.56 (0.35–0.91) for poor sleep quality compared to those in their 20s. The prevalence ratio of the obesity group for poor sleep quality was 1.53 (1.10–2.12). The prevalence ratio of musculoskeletal pain group for poor sleep quality was 1.92 (1.29–2.84).

**Conclusions:**

Age, obesity and musculoskeletal pain were factors affecting the poor quality on sleep of daytime workers. In addition, work type related to short sleep duration.

## Background

Sleep is essential for physical and mental health. Deep sleep for a particular number of hours is important for maintaining a normal human life. Interest in sleep is growing in today’s world, as are the percentages of workers with sleep disorders those undergoing treatment for sleep disorders [1]. In a telephone survey of 3400 people aged 15 years or older in Korea, 17% of the respondents had sleep disorder symptoms [2]. According to another telephone survey of 5000 Korean adults, 22.8% of the respondents had sleep disorders [3].

Symptoms of sleep disorders include poor concentration, fatigue, anxiety, and disorientation. In particular, sleep disorders cause daytime sleepiness, reduce work efficiency, and increase the risk of accidents [4]. Sleep disorders contribute to the deterioration of an individual’s quality of life and decrease the efficiency of a social organization. As sleep disorders are becoming increasingly common, many studies have been conducted on this subject. Moreover, there have been many studies on sleep disorders in shift workers. In Korea, Kim [4], Kim [5], and Son [6] have all studied shift workers and noted that shift work is associated with sleep disorders [7]. In a study on insomnia among middle-aged individuals in the UK, 18.8% of the respondents had a sleep disorders and the risk of sleep disturbance was higher in the night shift group [8]. Night shift is known to be an important factor interfering with workers’ sleep. Previous studies on the sleep of workers was mostly about the risks of night shift.

There have been many researches about sleep disturbance in workers. However, there have not been many studies on the sleep quality of Korean daytime workers. It seems that a study focusing only on daytime workers that do not work at night and shift work is needed.

This study was conducted to identify the sleep quality and duration of daytime workers and the factors that impact their quality of sleep.

## Methods

### Subjects

This study investigated all workers of an electronic parts manufacturing workshop in Gumi, Gyeongsangbuk-do, Korea. Except for those responsible for security work, most worked during the daytime. After excluding 18 shift workers among the 1356 workers, 1338 daytime workers were investigated. After further excluding the 167 workers whose questionnaire responses were not detailed enough or lacking, 1171 were chosen as the final subjects. This study, conducted in June 2016, was approved by the institutional review board of Soonchunhyang University Hospital in Seoul, and the approval number is Medicine 2018–02.

### Questionnaire

This study used a self-administered questionnaire to investigate demographic variables, work type, working period, sleep duration and quality and musculoskeletal symptoms. Regular health checkup was conducted for the workers’ clinical examination and physical measurements.

#### Variables

For demographic characteristics, the factors considered were sex, age, alcohol drinking, smoking, and exercise. This study also conducted a health checkup for workers and performed a clinical examination of their height, weight, waist circumference, blood pressure, obesity status, blood sugar, and blood lipid levels. For metabolic syndrome, this study followed the criteria suggested in the National Cholesterol Education Program Expert Panel on Detection, Evaluation, and Treatment of High Blood Cholesterol in Adults (Adult Treatment Panel III) [9]. To assess symptoms of musculoskeletal pain, this study used a questionnaire from the musculoskeletal risk factors survey guidelines in the Korea Occupational Safety and Health Agency’s KOSHA CODE H-9-2016 [10].

#### Quality of sleep assessment

The PSQI-K, a Korean version of the Pittsburgh Sleep Quality Index (PSQI) developed by the University of Pittsburgh, was used to measure the quality of sleep. In a study comparing the Korean version with the original PSQI to assess quality of sleep, the PSQI-K was determined to be reliable and valid [11]. The PSQI is a self-administered questionnaire that assesses the quality of sleep for a month. There are 19 questions belonging to 7 sub-categories. Each sub-category is rated on a scale of 0 to 3, and the total score for the sub-categories is 21 points. Higher scores indicate a lower quality of sleep. Sensitivity and specificity were found to be high when the total PSQI score exceeded the cut-off of 6 points. Hence, those with 6 or more points were categorized into the poor sleep group while those with 5 or fewer points were categorized into the good sleep group [12]. The 7 sub-categories of the PSQI are as follows: subjective sleep quality, sleep latency, sleep duration, habitual sleep efficiency, sleep disturbance, sleep medication use, and daytime dysfunction. Each category is rated from 0 to 3 points. To analyze the sub-categories that assess quality of sleep, this study categorized those who scored 0 or 1 point as those without sleep-related symptoms, and those with 2 or 3 points as those with sleep-related symptoms.

### Analyses

#### Classification of variables

In this study, workers’ alcohol drinking per week was studied. They were divided into 3 groups: non-drinkers, 1 drink per week, and over 2 drinks per week. For smoking, this study categorized the subjects into non-smokers, ex-smokers, and current-smokers. Those who exercised at a moderate or intense level or walked 5 times per week were categorized as exercising workers and those who did not as non-exercising workers. For working period, the subjects were divided into 4 groups: 5 or fewer years, 6–10 years, 11–15 years, and 16 or more years. For work type, the subjects were divided into 3 groups: blue-collar worker, office worker, and researcher. Blue-collar is a worker working in electronics production and they work with manufacturing management, facility management and quality control. Office worker is in charge of planning, relations, finance, personnel, education and general works of company. A researcher is a worker who carries out product development, technology research and problem solving of product. In each type of work, the role and job description are different and the strength and stress of work are different.

The subjects who met Standard 2 (pain in one or more areas that has continued for more than a week or occurs at least once per month at a moderate or high level) classified by the US National Institute for Occupational Safety and Health were defined as those with musculoskeletal symptoms [13].

This study defined 6 or fewer hours of sleep as a short sleep duration. Studies on number of sleeping hours have defined the lack of sleep in various ways, including 7 h or fewer, 6 h or fewer, or 5 h or fewer; there is no consensus on the exact short sleep duration [14]. In general, however, many studies have categorized 6 h or fewer as lack of sleep, and that is the definition used in this study [15, 16].

#### Statistical analyses

This study performed a t-test and analysis of variance (ANOVA) to investigate the correlations the following independent variables: demographic characteristics, working period, work types, metabolic syndrome and musculoskeletal symptoms. The dependent variables were sleep duration and PSQI score. Chi-square was performed through the analysis of variables and sleep duration (fewer than 6 h), variables and poor sleep quality. As a result, variables with *p*-value of less than 0.3 of both were selected and included in the calculation of prevalence ratio (PR). We included age, sex as independent variables which expected to have biologically significant relationship. To analyze the factors that contribute to short sleep duration, sleep quality, and the 7 sub-categories of the PSQI, this study used a Cox regression analysis and calculated PR and 95% confidence intervals (CI). A previous studies on method of prevalence ratio in cross-sectional study, Cox regression and Poisson regression analysis are more interpretable and easier to communicate to non-specialist than logistic regression [17, 18]. Cox regression used in this study because there were less serious problems than in the other methods in calculating PR [19]. We selected variable that result of Cox regression analysis about sleep quality. Variables with significant PR of Cox regression model were selected to analyze sub-categories of PSQI. In the Cox regression model, the variables were analyzed by the enter method.

SPSS 18.0 (IBM Corp., Armonk, NY, USA) was used for all analyses, and statistical significance was defined as *p* < 0.05.

## Results

With respect to sleep duration, the researchers had 6.17 h of sleep, which was significantly lower than that of blue-collar (6.42 h) and office worker (6.38 h). This study categorized sleep duration of 6 or fewer hours per day as short sleep duration. The number of researchers in the short sleep duration was 116 (57.4%), which was higher percentage than that of blue-collar was 262 (44.9%), office worker was 152 (52.2%). The number of those 50s or older age in the short sleep duration was 87 (57.6%), of those in their 40s was 144 (53.9%), in their 30s was 232 (44.4%), and in their younger than 30 was 102 (49.3%). The number of metabolic syndrome in short sleep duration was 83 (58.0%), which was higher percentage than that of normal group 477 (47.7%) (Table 1).

**Table 1:** General and occupational characteristics of study subjects by sleep duration

Variables	Sleep duration		Sleep duration		
	Mean ± SD	*p*-value^a^	Fewer than 6 h	Over than 6 h	
			n (%)	n (%)	*p-*value^b^
Age (in years)		0.384			0.009
< 30	6.37 ± 1.03		102 (49.3)	105 (50.7)	
30–39	6.41 ± 0.96		232 (44.4)	291 (55.6)	
40–49	6.32 ± 0.97		144 (53.9)	123 (46.1)	
≧50	6.27 ± 1.09		87 (57.6)	64 (42.4)	
Sex		0.570			0.871
Male	6.36 ± 0.95		516 (49.1)	534 (50.9)	
Female	6.42 ± 1.32		49 (50.0)	49 (50.0)	
Working period (years)		0.502			0.776
≦5	6.43 ± 0.92		110 (47.2)	123 (52.8)	
6–10	6.44 ± 0.99		74 (42.3)	101 (57.7)	
11–15	6.38 ± 0.97		76 (46.6)	87 (53.4)	
≧16	6.57 ± 1.10		39 (44.8)	48 (55.2)	
Work type		0.006			0.004
Blue-collar	6.42 ± 0.94		262 (44.9)	322 (55.1)	
Office worker	6.38 ± 1.09		152 (52.2)	139 (47.8)	
Researcher	6.17 ± 0.94		116 (57.4)	86 (42.6)	
Smoking		0.171			0.580
Non-smoker	6.42 ± 1.02		230 (46.8)	261 (53.2)	
Ex-smoker	6.44 ± 1.05		116 (47.5)	128 (52.5)	
Current-smoker	6.31 ± 0.89		172 (50.4)	169 (49.6)	
Alcohol drinking		0.703			0.481
Non-drinker	6.42 ± 1.04		57 (44.9)	70 (55.1)	
1/week	6.40 ± 0.97		241 (47.3)	269 (52.7)	
Over 2/week	6.36 ± 0.99		220 (50.2)	218 (49.8)	
Exercise		0.187			0.238
No	6.41 ± 1.00		332 (46.9)	376 (53.1)	
Yes	6.33 ± 0.95		186 (50.7)	181 (49.3)	
BMI (kg/m^2)c^		0.308			0.186
< 23	6.43 ± 0.97		179 (44.9)	220 (55.1)	
23–25	6.37 ± 0.99		152 (49.7)	154 (50.3)	
25–30	6.30 ± 1.00		204 (52.4)	185 (47.6)	
≧30	6.30 ± 1.14		25 (52.1)	23 (47.9)	
Metabolic syndrome		0.115			0.021
No	6.38 ± 0.96		477 (47.7)	522 (52.3)	
Yes	6.24 ± 1.20		83 (58.0)	60 (42.0)	
Musculoskeletal Pain		0.058			0.126
No	6.39 ± 0.96		430 (48.2)	463 (51.8)	
Yes	6.24 ± 1.11		100 (54.3)	84 (45.7)	

^a^Calculated using t-test and ANOVA

^b^Calculated using chi-square test

^c^*BMI* Body Mass Index

In the PSQI, women’s score was 5.48 points, which was significantly higher than that of men, at 4.40. The PSQI score of the musculoskeletal pain group was 5.50 points, which was higher than that of the group without musculoskeletal pain, at 4.32. The number of musculoskeletal pain in the poor sleep quality was 34 (45.9%), which was higher percentage than that without musculoskeletal pain 105 (23.2%). Sleep quality tended to better as the workers got older. The number of those younger than 30 in the poor sleep quality was 39 (37.1%), of those in their 30s was 70 (28.8%), in their 40s was 29 (20.3%), and in their 50s or older was 12 (16.9%). As BMI increased, the percentage of poor sleep quality increased. Those BMI of 30 or higher in the poor sleep quality was 9 (40.9%), in their 25–30 was 64 (32.5%), in their 23–25 was 42 (25.1%), and in their fewer than 23 was 35 (20.1%) (Table 2).

**Table 2:** General and occupational characteristics of study subjects by sleep quality

Variables	PSQI score^a^		Sleep quality		
	Mean ± SD	*p*-value^b^	Good	Poor	
			n (%)	n (%)	*p-*value^c^
Age (in years)		0.059			0.005
< 30	4.81 ± 2.53		66 (62.9)	39 (37.1)	
30–39	4.60 ± 2.25		173 (71.2)	70 (28.8)	
40–49	4.24 ± 1.95		114 (79.7)	29 (20.3)	
≧50	4.04 ± 2.11		59 (83.1)	12 (16.9)	
Sex		0.003			0.082
Male	4.40 ± 2.18		386 (74.2)	134 (25.8)	
Female	5.48 ± 2.57		26 (62.9)	16 (38.1)	
Working period (years)		0.219			0.610
≦5	4.33 ± 2.02		79 (71.8)	31 (28.2)	
6–10	4.67 ± 2.48		54 (68.4)	25 (31.6)	
11–15	5.05 ± 2.59		48 (64.0)	27 (36.0)	
≧16	4.56 ± 2.15		29 (74.4)	10 (25.6)	
Work type		0.463			0.290
Blue-collar	4.52 ± 2.34		203 (71.0)	83 (29.0)	
Office worker	4.31 ± 2.05		113 (77.9)	32 (22.1)	
Researcher	4.65 ± 2.06		71 (74.7)	24 (25.3)	
Smoking		0.428			0.881
Non-smoker	4.56 ± 2.36		174 (73.1)	64 (26.9)	
Ex-smoker	4.26 ± 2.00		97 (74.6)	33 (25.4)	
Current-smoker	4.55 ± 2.25		113 (72.0)	44 (28.0)	
Alcohol drinking		0.145			0.593
Non-drinker	4.37 ± 2.21		47 (75.8)	15 (24.2)	
1/week	4.42 ± 2.14		194 (74.3)	67 (25.7)	
Over 2/week	4.94 ± 2.25		142 (70.6)	59 (29.4)	
Exercise		0.437			0.266
No	4.54 ± 2.23		251 (74.7)	85 (25.3)	
Yes	4.38 ± 2.27		132 (70.2)	56 (29.8)	
BMI (kg/m^2)d^		0.081			0.021
< 23	4.25 ± 2.06		139 (79.9)	35 (20.1)	
23–25	4.35 ± 2.07		125 (74.9)	42 (25.1)	
25–30	4.73 ± 2.37		133 (67.5)	64 (32.5)	
≧30	5.14 ± 3.01		13 (59.1)	9 (40.9)	
Metabolic syndrome		0.135			0.415
No	4.43 ± 2.16		358 (73.8)	127 (26.2)	
Yes	4.84 ± 2.59		52 (69.3)	23 (30.7)	
Musculoskeletal pain		0.000			0.000
No	4.32 ± 2.15		347 (76.8)	105 (23.2)	
Yes	5.50 ± 2.31		40 (54.1)	34 (45.9)	

^a^*PSQI* Pittsburgh Sleep Quality Index

^b^Calculated using t-test and ANOVA

^c^Calculated using chi-square test

^d^*BMI* Body Mass Index

The PR of researcher group for short sleep duration was 1.27 (95% CI: 1.02–1.58), which was higher than that of blue-collar and office worker.

Those with a PSQI score of 6 or more were categorized into the poor sleep quality group, and the variables that had significant PRs included age, BMI of 25 or higher and musculoskeletal pain. For age, compared to those younger than 30, the PR of those in the 50s or older for poor sleep quality was 0.47 (0.25–0.91), and of those in their 40s was 0.56 (0.35–0.91). Compared to those with BMI lower than 25, the PR of those with BMI of 25 or higher for poor sleep quality was 1.53 (1.10–2.12). The PR of musculoskeletal pain for poor sleep quality was 1.92 (1.29–2.84) (Table 3).

**Table 3:** Prevalence ratio (PR) of factors related to short sleep duration and sleep quality

Variables	Short Sleep duration (fewer than 6 h)		Sleep quality (poor)	
	PR^a^	95% CI	PR	95% CI
Age (in years)				
< 30	1.00		1.00	
30–39	0.90	0.72–1.14	0.79	0.53–1.17
40–49	1.10	0.85–1.43	0.56	0.35–0.91
≧50	1.18	0.88–1.58	0.47	0.25–0.91
Sex				
Male	1.00		1.00	
Female	1.07	0.80–1.45	1.30	0.77–2.20
Work type				
Blue-collar	1.00		1.00	
Office worker	1.12	0.91–1.38	0.86	0.57–1.32
Researcher	1.27	1.02–1.58	0.90	0.57–1.43
Exercise				
No	1.00		1.00	
Yes	1.07	0.89–1.29	1.21	0.86–1.71
BMI (kg/m^2^)^b^				
< 25	1.00		1.00	
≧25	1.14	0.96–1.36	1.53	1.10–2.12
Metabolic syndrome				
No	1.00		1.00	
Yes	1.18	0.93–1.50	1.36	0.87–2.15
Musculoskeletal pain				
No	1.00		1.00	
Yes	1.16	0.93–1.45	1.92	1.29–2.84

^a^*PR* Prevalence Ratio by using Cox regression analysis

^b^*BMI* Body Mass Index

This study performed a Cox regression analysis with the 7 sub-categories of the PSQI as dependent variables. For age, subjective sleep quality and daytime dysfunction were related. Compared to those in their 20s, the PR of those in their 40s was 0.51 (0.33–0.81), and of those in the 50s or older was 0.34 (0.17–0.65) for subjective sleep quality. With respect to daytime dysfunction, compared to those in their 20s, the PR of those in their 30s was 0.59 (0.40–0.86), in their 40s was 0.28 (0.16–0.51), and in their 50s was 0.09 (0.03–0.30). Compared to men, the PR of women for subjective quality of sleep was 1.97 (1.37–2.85), for sleep latency was 1.57 (1.03–2.40), and for habitual sleep efficiency was 2.68 (1.13–6.33). The PR of musculoskeletal pain group was 2.16 (1.57–2.98) for subjective sleep quality, 1.54 (1.09–2.17) for sleep latency, 9.28 (2.92–29.5) for sleep disturbance, and 2.09 (1.41–3.10) for daytime dysfunction (Table 4).

**Table 4:** Prevalence ratio of factors related to the 7 sub-categories of the PSQI^a^

Variables	PR^b^ (95% CI)						
	Subjective sleep quality	Sleep latency	Sleep duration	Habitual sleep efficiency	Sleep disturbances	Sleep medication	Daytime dysfunction
Age (in years)							
< 30	1.00	1.00	1.00	1.00	1.00	1.00	1.00
30–39	0.87 (0.62–1.22)	0.94 (0.65–1.35)	0.90 (0.72–1.14)	0.83 (0.35–1.93)	0.00 (0.00–0.00)	0.20 (0.02–2.24)	0.59 (0.40–0.86)
40–49	0.51 (0.33–0.81)	0.75 (0.48–1.18)	1.10 (0.85–1.43)	0.69 (0.24–2.02)	0.00 (0.00–0.00)	1.71 (0.30–9.77)	0.28 (0.16–0.51)
≧50	0.34 (0.17–0.65)	0.92 (0.55–1.53)	1.18 (0.88–1.58)	0.21 (0.03–1.71)	0.00 (0.00–0.00)	0.75 (0.06–8.71)	0.09 (0.03–0.30)
Sex							
Male	1.00	1.00	1.00	1.00	1.00	1.00	1.00
Female	1.97 (1.37–2.85)	1.57 (1.03–2.40)	1.07 (0.80–1.45)	2.68 (1.13–6.33)	0.00 (0.00–0.00)	1.96 (0.22–17.3)	1.41 (0.87–2.28)
BMI (kg/m^2^)^c^							
< 25	1.00	1.00	1.00	1.00	1.00	1.00	1.00
≧25	1.09 (0.82–1.47)	1.07 (0.80–1.43)	1.14 (0.96–1.36)	1.48 (0.72–3.06)	1.52 (0.49–4.74)	1.16 (0.27–4.94)	0.88 (0.61–1.27)
Musculoskeletal pain							
No	1.00	1.00	1.00	1.00	1.00	1.00	1.00
Yes	2.16 (1.57–2.98)	1.54 (1.09–2.17)	1.15 (0.93–1.45)	0.79 (0.29–2.13)	9.28 (2.92–29.5)	4.51 (0.95–21.3)	2.09 (1.41–3.10)

^a^*PSQI* Pittsburgh Sleep Quality Index

^b^*PR* Prevalence Ratio by using Cox regression analysis

^c^*BMI* Body Mass Index

## Discussion

This study investigated the relationship among demographic characteristics, working period, work type, metabolic syndrome, musculoskeletal symptoms and sleep of daytime workers. The mean sleep duration was 6.36 h and mean PSQI score was 4.46. Work type was related to sleep duration. Age, BMI and musculoskeletal pain were significantly related to sleep quality.

In this study, daytime workers’ mean sleep duration was 6.36 h. This is lower than the mean sleep duration of 6.78 h of the 17,638 people who provided their data in the Korean National Health and Nutritional Examination Survey [20]. A study on 1238 daytime workers found a mean sleep duration of 6.58 h [21]. In a study of sleep duration and subclinical arterial disease, there was a low risk of cardiovascular disease at 7 h of sleep per day [22]. The mean sleep duration for subjects in this study was shorter than that sleep duration. This lack of sleep hours could be a risk factor for coronary heart disease, diabetes mellitus [23], and hypertension [24]. The average PSQI score in this study was 4.46 points: men scored 4.4 and women scored 5.48. In a study with 1008 workers in a manufacturing workshop, men scored 4.15 and women scored 4.77 on the PSQI. The scores in this study were higher than those in that study [25]. The average PSQI scores in a study with 2144 adults were also similar to those in this study, where men scored 4.37 and women scored 5.74 [26].

Sleep duration had no statistically significant association with age. Sleep duration has been known to decrease as age increases. Additionally, in this study, the sleep duration of those in their 20s was 6.37 h, while that of those in their 50s or older was 6.27 h. In this univariate analysis, as age increased, the group of short sleep duration was significantly increased. But, age was not significantly related to sleep duration in multivariate analysis. A population-based study of 1042 adults in Brazil reported that as age increased, there was a decrease in sleep efficiency, the percentage of rapid eye movement sleep, and slow wave sleep [27]. A meta-analysis of sleep studies showed that sleep duration and efficiency reduced as age increased [28]. Since this study was conducted on relatively younger workers, the age gap was not as large as in other studies, which might be why there was no significant decrease in sleep duration with an increase in age.

Sleep quality significantly increased as age increased. Compared to those in their 20s, the PR of those in their 50s or older for poor sleep quality was 0.47. In addition, compared to the 7 PSQI sub-categories, subjective sleep quality improved and daytime dysfunction decreased as age increased. Buysse and the other PSQI developers found that age was associated with subjective sleep quality and daytime dysfunction [12], which is consistent with the results of this study. Therefore, as age increased, the quality of sleep became better. This means that younger workers were more dissatisfied with their sleep and perceived that sleep limited their activities during the daytime. A sleep study of 5090 white-collar workers in Japan found that the percentage of those with sleep disorders decreased as age increased, which was consistent with this study’s results [29]. Meanwhile, a cohort study of 2406 adults in the UK found that the older group was more likely to have poor sleep quality, which contradicts this study’s findings. The cohort study assessed depression after retirement, reduced energy, and poor mental health as major causes of sleep disorders [30]. This study was conducted only with workers who are still working. Thus, their age was relatively low compared to those in the cohort study, and there were no retired individuals. These differences may account for the fact that this study had different results than the cohort study [31].

Researchers had shorter sleep duration than blue-collar and office worker. Depending on the type of work, there are differences in working conditions and types of job stress. Blue-collar workers reported being stressed owing to the physical environment, interpersonal conflicts, and job insecurity, whereas researchers had a high level of stress owing to continuous technology development and research activities, problem-solving, job demands, organizational injustice, and occupational climate [32]. Researchers’ sleep duration may be relatively shorter as a result of these differences in job stress factors.

Obesity is an additional factor that influences daytime workers’ sleep. Group with BMI of 25 or higher had no statistically significant association with short sleep duration. But, those with a BMI of 25 or higher had a 1.53 times greater risk for poor sleep quality than those with a BMI lower than 25. Meta-analysis of 45 cross-sectional or prospective studies of adults or children found a pooled obesity-short sleep OR of 1.6 for adults [33]. A study of 2006 young adults found that the risk of short sleep duration increased in the overweight and obese male group [34]. A study on sleep and obesity found that obesity had a significant correlation with sleep quality, more specifically with subjective sleep quality, duration, disturbance, and daytime dysfunction [35]. In this study, obesity had a significant association with poor sleep quality but, no significant association with PSQI sub categories. It is well known that obesity is associated with risk factors such as cardiovascular disease, cerebrovascular disease, hypertension, and diabetes mellitus. This study was able to identify that obesity was related to sleep quality.

This study found that workers with musculoskeletal pain had shorter sleeping hours and were more likely to have poor sleep quality. In this study, those with musculoskeletal pain were 1.92 times more likely than others to have poor sleep quality. A previous study on musculoskeletal pain and sleep found that the former was significantly related to short sleep duration and decreased sleep efficiency [36]. A quality of sleep study of 1650 patients with acute back pain revealed that the quality of sleep dropped by one step when the pain level doubled [37]. A study of 657 firefighters found that those with musculoskeletal pain were 2.89 times more likely than those without such pain to have poor sleep quality, which was consistent with this study’s results [38]. Among other sub-categories of the PSQI, musculoskeletal pain had a significant association with subjective sleep quality, sleep latency, sleep disturbance, and daytime dysfunction. A study of 40 people with shoulder impingement syndrome found that shoulder pain had a significant association with subjective sleep quality, latency, duration, efficiency, and disturbance [39]. A study of 1147 adolescents with musculoskeletal pain and sleep, poor subjective sleep perception was found to be associated with chronic musculoskeletal regional pain. [40]. Musculoskeletal pain can affect a poor subjective sleep quality and causes sleep disturbance due to persistent pain. Poor sleep quality can make musculoskeletal pain worse, which can cause a vicious cycle of pain and sleep disturbances.

Many previous studies on workers’ sleep disorders have focused on day-night shift workers and compared the sleep status of shift and non-shift workers. This study investigated only daytime workers. This has important implications in that there are few studies on sleep quality and quantitative analysis PSQI of Korean daytime workers.

This study has the following limitations. First, being a cross-sectional study, it was not possible to determine causal relationships between the factors related to sleep duration and sleep quality. In the future, a follow-up study should be conducted to investigate the factors that affect daytime workers’ sleep. Second, the sleep quality of the workers could be affected by many physical and mental health conditions, as well as socio-demographic, occupational characteristics and environmental factors. We analyzed the relationships of worker’s medical conditions, such as hypertension, diabetes mellitus, hyperlipidemia and hepatic enzyme abnormalities, with sleep quality. However, none of these were found to be significantly related to sleep quality. Work-related variables in this study included only work type and working period. However, other factors such as job stress, residence environmental, marital status and other medical condition could be important factors affecting sleep quality. This was also a major limitation of this study. Therefore, additional study on job stress, environmental factor and other medical condition is needed. Third, this study was conducted with relatively young workers in only one electronics workshop. Thus, the results were limited in terms of generalizability to the entire population. Fourth, there was a possibility that responses to the questionnaire could have been overestimated or underestimated owing to limitations associated with the subjective nature of the self-administered questionnaire. More specifically, the possibility of overestimated responses to sleep disturbance and musculoskeletal pain cannot be entirely excluded.

## Conclusions

The factor that had an effect on daytime workers’ sleep duration were the type of work. The factors that affected their sleep quality were age, obesity and musculoskeletal pain. Sleep problems due to age and type of work are difficult to solve because they cannot be controlled. However, controlling body weight for daytime workers can improve sleep quality. Body weight loss is not only effective in preventing various diseases caused by obesity, but also is a way to solve sleep problems in obese workers. It is necessary to plan a program that can manage obesity in the workplace and to have a practical effect on the workers. It should also be able to control musculoskeletal pain of daytime workers. It is needed to conduct periodic investigations of musculoskeletal hazards for heavy-weight handled and repetitive workers. In addition, there is a need for program to treat musculoskeletal pain in the workplace.

## Abbreviations

BMI: Body mass index
PR: Prevalence ratio
PSQI: Pittsburgh Sleep Quality Index
